# ZnO Polymeric Composite Films for *n*-Decane Removal from Air Streams in a Continuous Flow NETmix Photoreactor under UVA Light

**DOI:** 10.3390/nano11081983

**Published:** 2021-07-31

**Authors:** Crissie D. Zanrosso, Sandra M. Miranda, Batuira M. da Costa Filho, Jonathan C. Espíndola, Diego Piazza, Vítor J. P. Vilar, Marla A. Lansarin

**Affiliations:** 1 Chemical Engineering Department, Federal University of Rio Grande do Sul, R. Ramiro Barcelos 2777, Porto Alegre 90035-007, Brazil; crissiedz@hotmail.com; 2 Laboratory of Separation and Reaction Engineering-Laboratory of Catalysis and Materials (LSRE-LCM), Department of Chemical Engineering, Faculty of Engineering of the University of Porto, Rua Dr. Roberto Frias, 4200-465 Porto, Portugal; sandra.miranda@fe.up.pt (S.M.M.); batuirafilho@yahoo.com.br (B.M.d.C.F.); jonathan.espindola@hotmail.com (J.C.E.); 3 School of Civil Engineering, Architecture and Urban Design, University of Campinas, Campinas 13083-852, Brazil; 4 Laboratory of Polymers, Center for Exact Sciences and Technology, University of Caxias do Sul, R. Francisco Getúlio Vargas 1130, Caxias do Sul 95070-560, Brazil; diego.piazza@ucs.br

**Keywords:** polymeric composites, photocatalysis, photocatalyst stability, NETmix photoreactor

## Abstract

Polymeric composite films have been explored for many photocatalytic applications, from water treatment to self-cleaning devices. Their properties, namely, thickness and porosity, are controlled mainly by the preparation conditions. However, little has been discussed on the effect of thickness and porosity of polymeric composite films for photocatalytic processes, especially in gas phase. In the present study, different preparation treatments of ZnO-based polymeric composite films and their effects on its performance and stability were investigated. The polymeric composites were prepared by solution mixing followed by non-solvent induced phase separation (NIPS), using poly(vinylidene fluoride) (PVDF) as the matrix and ZnO-based photocatalysts. Different wet thickness, photocatalyst mass, and treatments (e.g., using or not pore-forming agent and compatibilizer) were assessed. A low ZnO/PVDF ratio and higher wet thickness, together with the use of pore-forming agent and compatibilizer, proved to be a good strategy for increasing photocatalytic efficiency given the low agglomerate formation and high polymer transmittance. Nonetheless, the composites exhibited deactivation after several minutes of exposure. Characterization by XRD, FTIR-ATR, and SEM were carried out to further investigate the polymeric film treatments and stability. ZnO film was most likely deactivated due to zinc carbonate formation intensified by the polymer presence.

## 1. Introduction

Photocatalysis, a highly efficient, economic, and environmental-friendly technology, is an interesting approach for environmental remediation purposes as it offers a remarkable potential for pollutants oxidation/reduction and, consequently, environmental protection [1]. Research has mainly been focused on the development of advanced photocatalytic materials and their immobilization in inert supports or its incorporation in substrates (composite materials), towards a substantially higher photocatalytic activity and avoiding post-treatment steps, respectively [2,3]. It is worth noticing that, when using composite materials, the process must be optimized in order to mitigate possible decreases in photocatalytic efficiency (e.g., increased mass transfer resistance, low catalyst homogeneity distribution, and reduced photocatalyst surface area exposed to light) [4].

Thin films can be defined as a layer that extends along with any two directions and is restricted along the third direction, having a small thickness (from nanometers to a few micrometers). This dimension is the main advantage of thin-film technology, providing cost reduction and miniaturization of devices [5]. The properties of the films are mainly controlled by the film structure, reaction condition, and treatment method [6].

Considering the potential substrates/supports for catalysts, polymers are particularly interesting as they are chemically inert, mechanically stable with high durability, inexpensive, readily available, and hydrophobic [7,8]. In addition, some of these materials (e.g., PVDF and its copolymers) also present UV light and oxidative radical resistance [9].

Photoactive polymeric composites have been systematically tested in batch reactors. However, its performance on continuous flow mode operation has been less explored. In a previous study reported by our group [10], thin films prepared by fusion showed significantly reduced porosity compared to solution mixing films and, consequently, their photocatalytic effectiveness was also drastically reduced. Moreover, several studies have also reported the use of photoactive polymeric composites in different sectors, including (i) construction [11,12,13], (ii) water/wastewater treatment [14,15], (iii) food industry [16,17], (iv) biomedical industry [18,19,20], and (v) pharmaceutical industry [21], among others. However, to the best of our knowledge, there are no studies assessing the effect of the thickness and porosity of polymeric composite films for continuous gas-phase photocatalytic processes. Furthermore, an extensive review found no evaluations on the possible catalysts’ deactivation effects caused by continuous flow use, especially in photocatalysts other than TiO_2_.

Therefore, the main objective of the present work is to investigate the effect of the preparation treatments of ZnO-based polymeric composite films on its photocatalytic activity. ZnO-based materials have been receiving enormous attention because of their low cost, multifunctional properties, great versatility, and antibacterial and antifungal properties [22,23,24]. The photocatalytic activity of the different films was assessed towards *n*-decane removal, as a model volatile organic compound (VOC), in gas phase using a continuous flow NETmix photoreactor irradiated by UVA LEDs. The polymeric composites were prepared by solution mixing followed by non-solvent-induced phase separation (NIPS) using poly(vinylidene fluoride) (PVDF) as the matrix and different ZnO-based photocatalysts (i.e., ZnO composites with compatibilizer (PAZ), with pore-forming agent (PVZ) or without treatment (PZ)). Photocatalyst deactivation was also assessed. XRD, FTIR-ATR, and SEM analysis were performed in order to evaluate changes on the polymeric matrix after exposition to UVA light. Furthermore, the effect of film wet thickness (50–150 μm), film photocatalyst mass (0–540 mg), use of a pore-forming agent (polyvinylpyrrolidone K30 (PVP)), and compatibilizer ((3-aminopropyl)triethoxysilane (APTES)/citric acid (CA)) was assessed.

## 2. Materials and Methods

### 2.1. Chemicals

The following reagents were used without further treatment: *n*-decane (Merck, purity ≥ 94%) was used as model VOC, zinc oxide (ZnO; Merck, Darmstadt, Germany), (3-aminopropyl)triethoxysilane (APTES; Sigma-Aldrich, St. Louis, MO, USA), citric acid (CA; (Sigma-Aldrich), polyvinylpyrrolidone K30 (PVP; Synth, São Paulo, Brazil), Triton X-100 (Sigma-Aldrich), N,N-dimethylacetamide (DMAc; Neon, São Paulo, Brazil), and ethanol (Dinâmica, São Paulo, Brazil). Deionized water was used throughout the experiments. Poly(vinylidene fluoride) (PVDF; Kynar grade 720) was dried at 70 °C overnight before use. Helium N50, nitrogen N50, and synthetic air N50 (O_2_: 20 ± 1%; H_2_O: <3 ppm; C_n_H_m_: <0.1 ppm; CO_2_: <1 ppm; CO: <1 ppm) were acquired from Air Liquide (Porto, Portugal).

### 2.2. Photocatalyst Preparation

Besides the commercial ZnO nanoparticles, in order to improve compatibility with the polymeric matrix, treated ZnO nanoparticles (A-ZnO) were also used as photocatalyst in this study. The treatment procedure was adapted from Ardeshiri et al. (2018) [25]. A total of 2 g of commercial ZnO and 4 mL of APTES were dispersed in 100 mL of ethanol. The suspension was stirred with reflux overnight at 70 °C. After centrifugation, the ZnO-APTES was washed with ethanol and dried at 50 °C for 24 h. A total of 1 g of ZnO-APTES and 0.52 g of citric acid were added to 30 mL of DMAc (promoting the reaction between the amine groups and the carboxylic acid). The solution was refluxed and stirred at 100 °C overnight. Subsequently, the nanoparticles were washed with ethanol and water, interspersed with centrifugation. Finally, the A-ZnO photocatalyst was dried at 50 °C for 24 h.

### 2.3. Catalyst Film Preparation

Initially, as a reference test, ZnO nanoparticles were immobilized directly onto the borosilicate glass slab of the NETmix window according to the methodology proposed by da Costa Filho, Araujo [26]. A solution containing 2 wt % photocatalyst suspension and approximately 50 µL of Triton X-100 was sonicated for 10 min at 50 kHz and, after that, sprayed on one side of the borosilicate glass slab, which was continuously heated at 150 °C on a heating plate. After drying and cooling, the borosilicate slab was weighed, and this procedure was repeated until 0.50 ± 0.02 g of nanoparticles was uniformly deposited.

**Figure 1:**
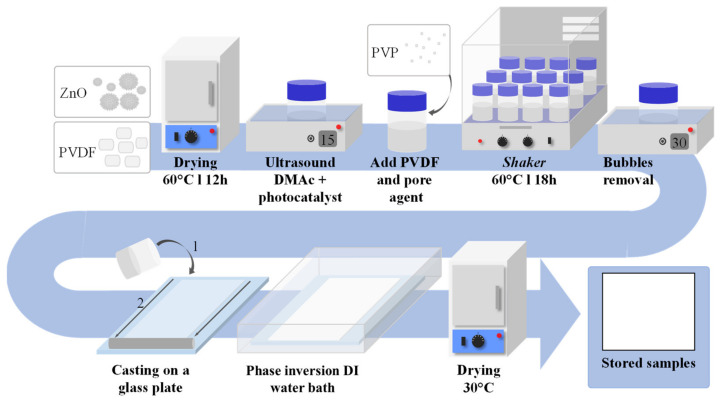
Polymeric composite films preparation scheme.

The polymeric PZ, PVZ, and PAZ films were prepared by solution mixing followed by NIPS [27]. Selected amounts of photocatalyst and 10 mL of DMAc were sonicated for 15 min. The polymer PVDF and pore-forming agent PVP (when used) were added to the mixture and kept in a shaker at 60 °C for 18 h. The samples were sonicated for 30 min and spread on a glass plate at a controlled wet thickness. Immediately afterward, the samples were immersed in a deionized water bath for film precipitation. After 24 h in deionized water, the films were dried at 30 °C. The preparation scheme is presented in Figure 1. It is important to note that in the preparation of polymeric films by the NIPS method, the independent control variable related to film thickness is the wet thickness, which is the spreading thickness of the solubilized mixture. In this structure, the solvent volume is still present, in addition to the solubilized polymer and other solids. After phase inversion, different dry thicknesses, as well as porous structure, were obtained as a function of the remaining solids and preparation conditions. Prior to testing, samples were cut at exactly size of the reactor window. Table 1 shows the catalyst films preparation compositions and photocatalyst mass of cut samples.

### 2.4. Catalyst Film Characterization

The Brunaeur–Emmett–Teller specific surface area (S_BET_) was calculated using N_2_ adsorption isotherms (NOVA 4200e, Quantachrome, Boynton Beach, FL, USA). The X-ray diffractograms (DRX) (D2 phaser Bruker, Billerica, MA, EUA) were obtained in the 2θ range from 10 to 80°, with a Kα copper X-ray generating source, voltage of 30 kV, fixed reading time of 1 s, and angular increment of 0.02°. The chemical structures were observed by Fourier transform infrared spectroscopy using a universal attenuated total reflectance sensor (FTIR-ATR) (Thermo Scientific Nicolet, Waltham, MA, USA) in the range 600 to 4000 cm^−1^. The samples were analyzed after the photocatalytic test to check for possible changes in the crystalline and chemical structures.

FTIR-ATR results were also used to quantify the electroactive phase content of PVDF [28]. The β-phase in a sample containing only α and β PVDF can be calculated according to Equation (1), when assuming that FTIR absorption follows the Lambert–Beer law.

(1) $$F\left(\beta \right)=\frac{A_{\beta }}{\left(K_{\beta }/K_{\alpha }\right)A_{\alpha }+A_{\beta }}$$

where *A_α_* and *A_β_* are the absorbance at 766 and 840 cm^−1^, respectively, which are the distinctive peaks of each phase; *K_α_* and *K_β_* are the absorption coefficients at the respective wavenumber, respectively corresponding to 6.1 × 10^4^ and 7.7 × 10^4^ cm^2^ mol^−1^ [28].

The radiation power transmitted though the borosilicate glass slab with and without the catalyst film was measured using an UV radiometer. Without the catalyst film, the UV power was 245.5 W m^−2^. The transmittance value (%) for catalyst films was calculated by the ratio between the UV power that passed through the borosilicate coated with the catalyst and without the catalyst (245.5 W m^−2^).

### 2.5. Photocatalytic Oxidation Tests

The NETmix photoreactor consists of a stainless-steel slab engraved with a network of cylindrical chambers interconnected by prismatic channels and sealed by a borosilicate glass window (irradiated area of 55.7 cm^2^). The photocatalytic films, previously cut according to the irradiated window dimensions, were assembled between the borosilicate and stainless-steel slabs, in contact with the air flow within the channels and chambers. The reactor presents two flow distributors with one inlet and eight outlets, located at the inlet and outlet of the reactor. One flow distributor was used to ensure the correct distribution of the feed gas stream through the network of 8 chambers. Another flow distributor was located at the end of the network, from where the outlet gas was fed to the VOC analytical system. A UVA LED plate (light source) with three series of 6 UVA LEDs (Roithner Lasertechnik; 900 mW per LED; maximum wavelength of 365 nm) was placed above the reactor window. A full and detailed description of the NETmix can be found in previous works [26,29,30].

The air stream generation system consists of three mass flow controllers (MFC, El-Flow, Bronkhorst High-Tech B.V., Ruurlo, the Netherlands) connected to compressed and synthetic air lines. MFC 1 and 3 were responsible for the control of the compressed air flow through two different Woulff bottles, containing deionized water and *n*-decane, producing the humid and *n*-decane saturated air streams, respectively. A synthetic air stream, controlled by the MFC 2, was used to adjust the *n*-decane concentration and humidity of the inlet feed air stream. The temperature of the Woulff bottles was kept constant (8.0 °C) using a thermostatic bath. Finally, the three air streams joined in a single feed air stream, which was continuously fed to NETmix reactor. The *n*-decane concentration was measured and monitored by an in-line gas chromatography analytical system (MGC Fast GC, Dani Instruments SpA, Cologno Monzese, IT), with a silica capillary column and flame ionization detector (FID). Humidity, temperature, and CO_2_ were also monitored using a probe (IAQ-Calc™ indoor air sensor, Shoreview, MN, USA).

The efficiencies of the different photocatalytic materials were assessed through considering the following experimental conditions: UVA irradiance = 245.5 W m^−2^, *n*-decane feed concentration (*C*_VOC,feed_) = 78.8 ppm, and total feed flow rate (*Q*_feed_) = 150 cm³ min^−1^.

Before turning on the LED system, we continuously fed the photoreactor until steady-state condition was achieved in the dark.

Conversion rate ($r_{\mathrm{VOC}}$, µmol min^−1^), conversion (%), mineralization (%), and apparent reaction rate ($r_{\mathrm{app}}$, µmol min^−1^ g^−1^) were calculated according to Equations (2)–(5), respectively.

(2) $$r_{\mathrm{VOC}}=\left(C_{\mathrm{VOC},\mathrm{feed}}-C_{\mathrm{VOC},\mathrm{exit}}\right)\times Q_{\mathrm{feed}}\;$$

(3) $$\mathrm{Conversion}=\left(1-\frac{C_{\mathrm{VOC},\mathrm{exit}}}{C_{\mathrm{VOC},\mathrm{feed}}}\right)\times 100\;$$

(4) $$\mathrm{Mineralization}=\left(\frac{C_{{\mathrm{CO}}_{2}}}{C_{\mathrm{VOC},\mathrm{feed}}}\times \frac{1}{n}\right)\times 100\;$$

(5) $$r_{\mathrm{app}}=\frac{\left(C_{\mathrm{VOC},\mathrm{feed}}-C_{\mathrm{VOC},\mathrm{exit}}\right)\times Q_{\mathrm{feed}}}{W}\;$$

where $C_{\mathrm{VOC}}$ and $C_{{\mathrm{CO}}_{2}}$ are the concentrations (µmol cm^−3^) of *n*-decane and CO_2_, respectively; *n* is the carbon number in *n*-decane molecule; $Q_{\mathrm{feed}}$ is the total feed flow rate (cm^3^ min^−1^); and *W* is the photocatalyst mass (g).

## 3. Results and Discussion

### 3.1. Photocatalyst Deactivation

The composite films stability in terms of photocatalytic activity was firstly evaluated for three different types of polymeric composite films (untreated ZnO—PZ-1, pore-forming agent addition—PVZ-1, and pore-forming agent combined with ZnO compatibilizer treatment—PAZ-1) at the same photocatalyst:polymer ratio (0.50:2.25). *n*-Decane photocatalytic oxidation (PCO) tests were performed in continuous mode for 5 h. For comparison purposes, a control experiment was carried out using the same amount of photocatalyst immobilized directly on the borosilicate glass slab (Z-1). In addition, the possible effect of an increase in photocatalyst mass was evaluated through an extra assay using the PVZ-4 composite film (photocatalyst:polymer ratio of 2.25:2.25).

**Figure 2:**
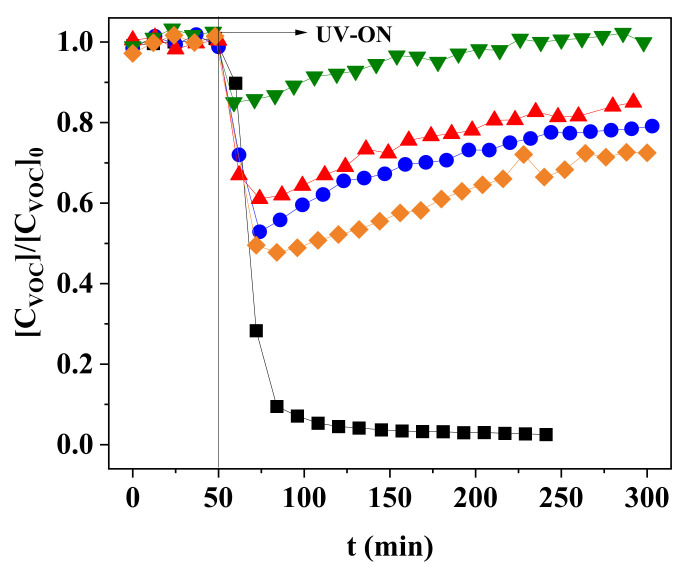
Profiles of *n*-decane removal by PCO using the NETmix photoreactor with different composite films: Z-1 (■), PAZ-1 (●), PVZ-1 (▲), PZ-1 (▼), and PVZ-4 (♦). Conditions: irradiation intensity = 245.5 W m^−2^; *C*_VOC,feed_ = 78.8 ppm; *Q*_feed_ = 150 cm^3^ min^−1^.

The concentration profiles, shown in Figure 2, suggest an apparent photocatalyst deactivation of the composite films over time. The deactivation was similar across all composite treatments and ranged from 45.3% to 65.5% reduction in activity over a 5 h reaction period. Liqiang et al. (2004) [31] reported similar activity loss when studying the heptane PCO using ZnO as photocatalyst. This loss was explained by zinc carbonate formation, resulting from adsorption of H_2_O, CO_2_, and CO on the catalyst surface, which induced deactivation in less than 18 h of use.

Contrary to that observed with the polymeric composite films, the Z-1 film did not exhibit deactivation, probably due to the greater number of active sites accessible for oxidation. Thus, if deactivation was taking place, this may not have been detected due to the availability of other active sites. In addition, the photocatalyst present in the polymeric films might have been deactivated either by polymeric matrix degradation or by the irreversible adsorption of degradation by-products in the polymeric matrix, preventing the contact of *n*-decane molecules with the active sites.

**Figure 3:**
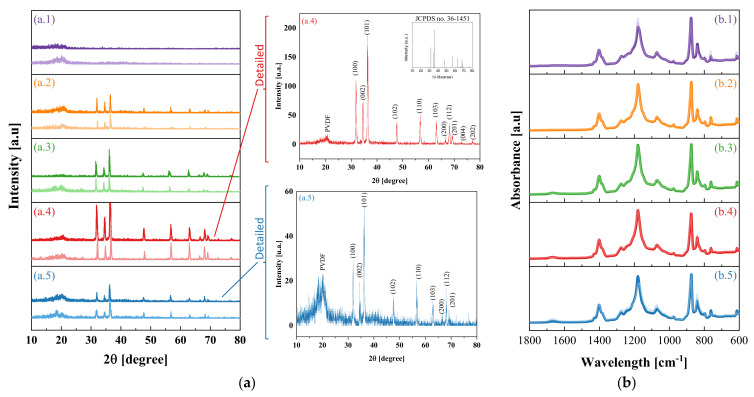
XRD (**a**) and FTIR-ATR (**b**) of samples (1) P-1, (2) PZ-1, (3) PVZ-1, (4) PVZ-4, and (5) PAZ-1, before (darker line) and after (lighter line) *n*-decane photocatalytic oxidation.

In order to identify damages on the composite films that could potentially explain the photocatalytic deactivation, we characterized the composite films after PCO in detail. In XRD analysis, the peaks observed for the hexagonal wurtzite ZnO (JCPDS 36-1451) were identified for all composites (Figure 3a). A signal between 18.5 and 26.5° was also observed, proving the polymer’s presence. PVDF is composed of both amorphous and crystalline phases that are distinguishable by performing both XRD and FTIR analysis. The stronger peaks associated with polymer phase were observed at 18.7° and 20.8°, which can be associated to α and β phase, respectively [28]. The strongest peak was at 36.3°, corresponding to ZnO.

After PCO tests, the composite films showed a negligible mass reduction, thereby confirming that the films were not severely damaged. However, the photocatalyst characteristic peak increased in the PZ-1 and PAZ-1 samples, which was probably caused by the degradation of the polymeric matrix covering the photocatalyst in these samples. Nonetheless, for samples PVZ-1 and PVZ-4, there was a reduction in the same peak, qualitatively indicating ZnO reduction in the sample, explained by photocatalyst leaching or corrosion.

For FTIR, all PVDF characteristic peaks and its electroactive phases α and β were observed (Figure 3b). The fraction of the crystalline β phase values, F[β], are summarized in Table 2. The degradation of the polymeric matrix is not clearly evident through reduction or appearance of new peaks in the spectrograph. However, the increase in F[β] for all composites indicates the transformation of α phase into β phase, of greater density, possibly associated with the degradation (albeit partial) of the polymeric matrix.

When investigating the degradation of PVDF using heavy ions of high energy, such as Au and Sm, Hossain et al. (2014) [32] concluded that the degradation mechanism consisted of homolytic cleavage or elimination of hydrogen and fluorine. Therefore, the formation of unsaturated bonds in the main carbon chain of the polymeric composite films in the present study is expected if degradation is taking place, which would result in an increase in the intensity observed at wavelengths 1752, 1711, and 1613 cm^−1^. It is noteworthy that the degradation observed by heavy ions is more intense than that resulting from the irradiation process with UVA light. Other studies have also found changes in the wavelengths 1600–1700 cm^−1^ in PVDF [33] and PVDF/TiO_2_ [34] exposed to 1710 h and 30 days of UV irradiation, respectively, which were also attributed to the formation of carbon double bonds generated by defluorination. Another important peak to confirm degradation is 678 cm^−1^, which represents changes due to polymeric chain defects, such as head-to-head configuration [33].

**Figure 4:**
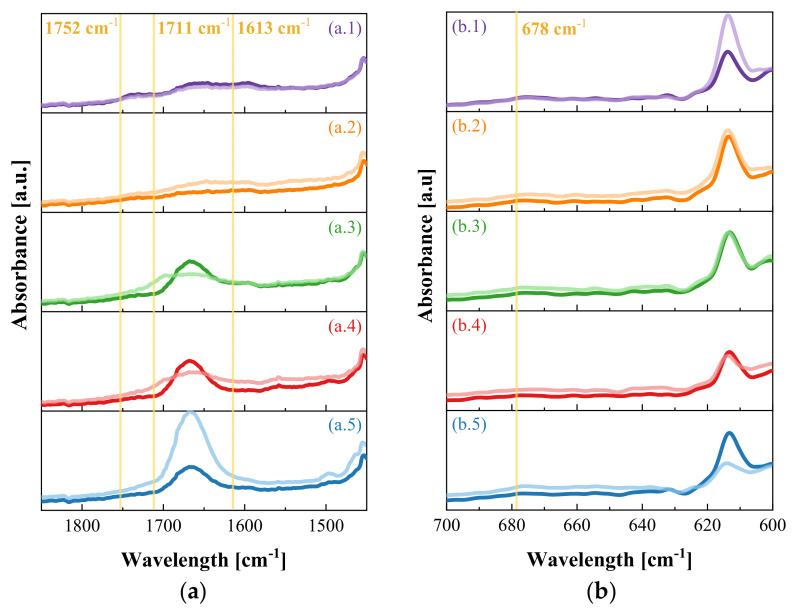
FTIR-ATR in the ranges (**a**) 1450-1850 and (**b**) 600-700 cm^−1^ of samples (1) P-1, (2) PZ-1, (3) PVZ-1, (4) PVZ-4, and (5) PAZ-1, before (darker line) and after (lighter line) *n*-decane photocatalytic oxidation.

Figure 4 shows a peak at 1666 cm^−1^, characteristic of the C=O bond of the PVP pore-forming agent and of modification with the compatibilizer APTES. Therefore, the wavelengths related to the degradation of PVDF can present interference, since, in the case of samples with PVP, apparently it was degraded throughout the exposure, while the sample with APTES showed an increase in intensity of the C=O bond, since nanoparticles covered with the compatibilizer agent are exposed on the surface.

To better compare the results, we used the relationship between the intensities after the photocatalytic assay and its untested pair at each peak associated with degradation (Table 3). The P-1 film, i.e., without photocatalyst, maintained these peak intensities, with values between 0.93 and 0.99 for all wavelengths. By contrast, all composites showed major differences after the photocatalytic test, especially for the wavelengths 1613 and 678 cm^−1^. Considering that a reduction in PVP took place, the increase due to the formation of unsaturated bonds in the polymer chain may have been even greater. This result is also related to the greater formation of the β phase, since to compensate for the imbalance caused to the polymer chain, for most polymers, there is an increase in crystallinity. In the case of PVDF, the stretching of the chains also generates a greater increase in the β phase, as shown in Table 2.

**Figure 5:**
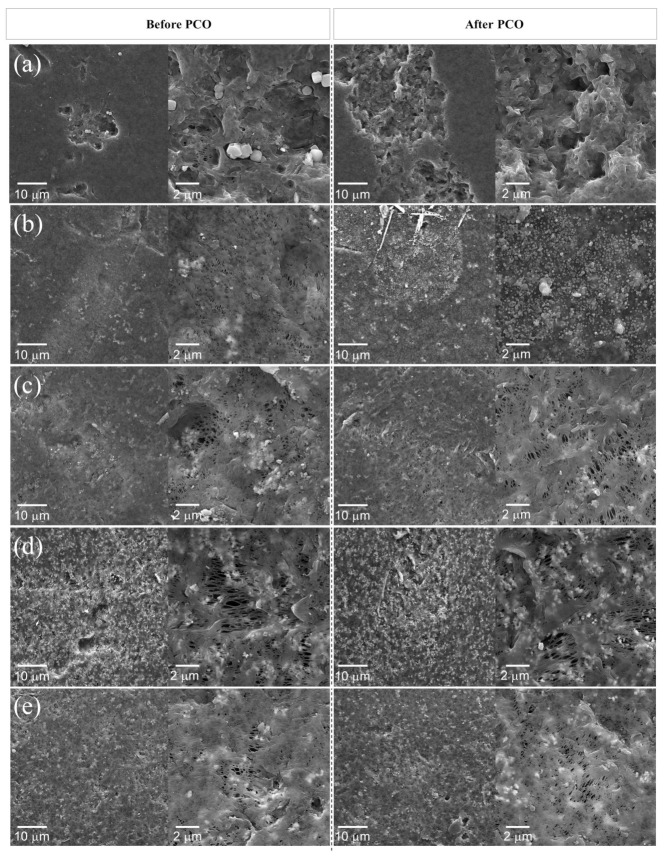
SEM images for samples before and after *n*-decane photocatalytic oxidation: (**a**) P-1, (**b**) PZ-1, (**c**) PVZ-1, (**d**) PVZ-4, and (**e**) PAZ-1.

The SEM images for all samples are shown in Figure 5, before and after the PCO test. For P-1, the disappearance of particles can be seen after the photocatalytic test. However, it is unclear as to whether these particles represent PVP that did not leave the matrix in the phase separation stage or contamination due to high material electrostatics. As previously mentioned, the PVP characteristic peak in FTIR-ATR was diminished after PCO, a reduction that could be related to these particles. Nevertheless, these particles were not observed for any of the other composites prepared with PVP, despite showing the same effect on FTIR analysis.

**Figure 6:**
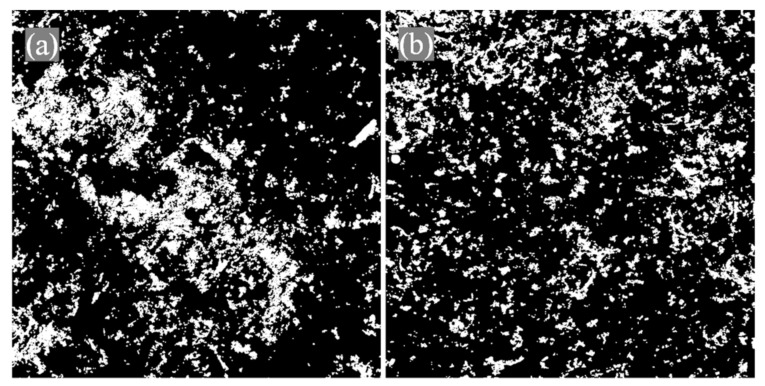
SEM images for (**a**) PVZ-1 and (**b**) PAZ-1 prior to *n*-decane photocatalytic oxidation.

The composites PVZ-1, PVZ-4, and PAZ-1 showed a qualitatively higher quantity of larger-sized pores in comparison with sample PZ-1. The higher amount of ZnO on the PVZ-4 sample surface is also disclosed by SEM analysis. Furthermore, as expected, the APTES treatment increased the ZnO particle dispersion in matrix, evidenced by comparing Figure 5c–e with image treatment (ImageJ binary transformation) in Figure 6. After PCO, no change was evident for composites prepared with PVP. However, formation of a new morphology on the PZ-1 sample surface was observed, indicating that some deposition of material took place during the PCO test. Notably, no formation of new peaks was revealed by other characterization techniques, such as XRD and FTIR-ATR, for this sample, rendering results about the nature of these deposits inconclusive.

SEM and FTIR-ATR results showed that the polymer was impaired after the PCO test. However, these changes were subtle and, unlike other reports in the literature, this did not cause qualitative problems in the material such as yellowing, fragility or mass loss. The XRD results suggest that ZnO photocorrosion may have also occurred, but this effect would not justify the intense activity loss observed. Furthermore, these alterations were not seen consistently across samples and there was no pattern for the different treatments, and thus the materials changes did not correlate to deactivation effects. These results suggest that the degradation most likely occurred due to deactivation caused by the adsorption of H_2_O, CO_2_, and CO, as previously mentioned, and intensified by the polymer presence.

### 3.2. Effect of Polymeric Films Preparation Procedures

**Figure 7:**
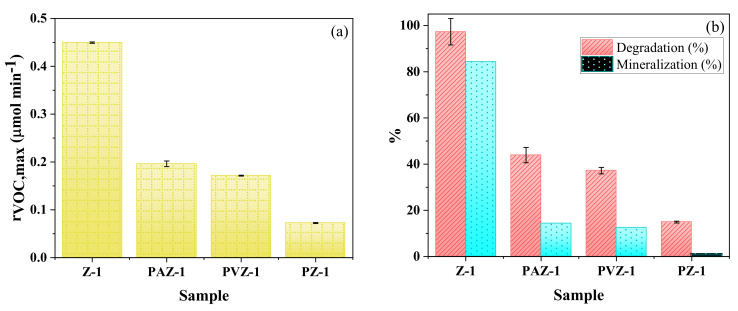
(**a**) Maximum *n*-decane conversion rate (*r_VOC,max_*) and (**b**) degradation and mineralization for samples Z-1, PAZ-1, PVZ-1, and PZ-1. Conditions: irradiation intensity = 245.5 W m^−2^; *C*_VOC,feed_ = 78.8 ppm; *Q*_feed_ = 150 cm^3^ min^−1^.

It is worth mentioning that, for the comparison purpose, all parameters presented in Table 4 were calculated at the maximum *n*-decane conversion rate achieved prior to deactivation. Photolysis experiment using the P-1 film, without photocatalyst, showed negligible removal of the *n*-decane (Table 4). Figure 7 shows that the use of a pore-forming agent during the preparation of the polymeric film improved the photocatalytic activity. As discussed previously, this improvement reinforces the hypothesis of a greater contact between *n*-decane molecules and photocatalyst in the pores present throughout the film thickness. The S_BET_ of samples with and without PVP were 9.007 and 7.007 cm^2^ g^−1^, respectively. This observed increase in surface area (27% increase), due to pore-forming agent inclusion, also supports the mentioned hypothesis.

The addition of APTES further increased *n*-decane removal efficiency. The compatibilizer improved the film stability due to the greater compatibility of ZnO, of hydrophilic origin, with PVDF, of hydrophobic origin. This pre-treatment (i) reduced the contact area between photocatalyst active sites and the polymer, decreasing polymeric matrix degradation, and (ii) increased nanoparticle adhesion, avoiding photocatalyst displacements. The greater photocatalytic activity obtained is associated with a better distribution of nanoparticles in the polymeric film, resulting from improved compatibility between the components [25]. In addition, the S_BET_ of PAZ-1 was 8.685 cm^2^ g^−1^, 26.5% greater than the sample PZ-1.

The photocatalyst thin film immobilized on the borosilicate glass slab showed better results when compared to the polymeric composite films. This can be mainly associated with the better exposure of the catalyst to UVA light, in addition to a lower mass transfer resistance when compared to the polymeric structure. However, this result may differ for other contaminants, since the polymer may favor the pollutant adsorption, resulting in higher photocatalytic oxidation rates [4].

### 3.3. Effect of Polymeric Composite Film Thickness

The PVDF and ZnO composition was previously optimized according to the greater porosity and photocatalytic efficiency in the methylene blue discoloration [27]. Although the processes in liquid and gas phases differ, it is expected that the composition of the selected composite film will provide appropriate photocatalytic activity also in gas phase, due to its largest exposed photocatalyst area when compared with the other evaluated compositions.

The NETmix photoreactor supplies radiation to the opposite side of the catalyst film in relation to the contaminant flow, i.e., back-side irradiation (BSI). Therefore, on the basis of literature results [26,30], with the increment on the catalyst film thickness, the reaction rate is expected to improve up to a maximum level, where the light is completely absorbed by the catalyst layer. A further increase on the catalyst film thickness will reduce the photocatalytic reaction rate since the charge carriers are generated far from the fluid–catalyst interface and consequently are more susceptible to recombination loss.

**Figure 8:**
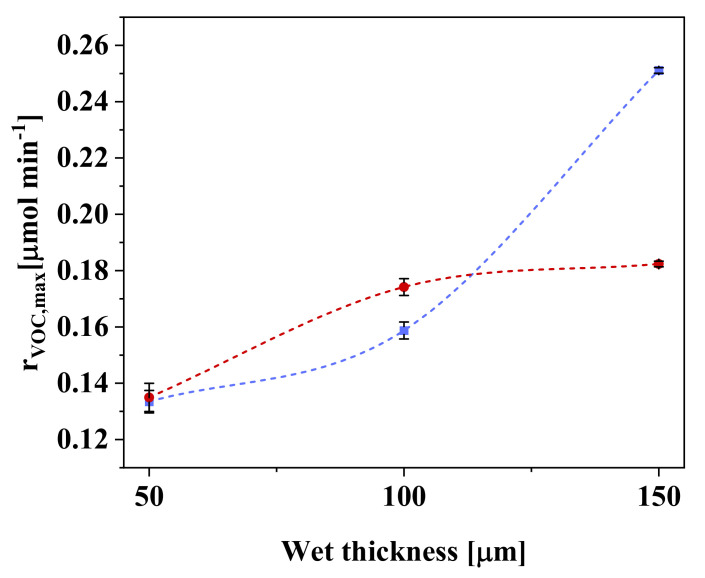
Maximum conversion rate of *n*-decane (*r*_VOC,max_) as a function of photocatalyst mass, with (■) and without (●) use of pore-forming agent. Conditions: irradiation intensity = 245.5 W m^−2^; *C*_VOC,feed_ = 78.8 ppm; *Q*_feed_ = 150 cm^3^ min^−1^.

Figure 8 shows the *n*-decane conversion rate as a function of the polymeric composite film thickness. For samples prepared without the pore-forming agent, an increase in the conversion rate was observed for film thickness between 50 and 100 µm, reflecting the greater amount of irradiated photocatalyst provided by the sample PZ-3. However, no effect was observed with a further increase from 100 to 150 µm.

However, the composite film prepared with the pore-forming agent (sample PVZ-4) showed a continuous increment on the *n*-decane conversion with an increase on the polymeric composite film from 50 to 150 µm. Thus, in comparison with PZ-3, an even higher conversion rate was observed for this sample, showing that the reaction occurred within the pores over the entire thickness of the film. This fact highlights the importance of the internal area in determining the conversion rate. In this particular case, the pore-forming agent enlarged the porous three-dimensional structure of the film, thereby leading to the higher removal rate due to the following factors: (i) higher porosity; (ii) better photocatalyst exposition; (iii) enhanced molecular diffusion within the film thickness, enabling the VOC molecules to reach the lower catalyst layers; and (iv) improved light distribution throughout the film. On the other hand, without the pore-forming agent (low porosity), these effects were much less substantial; hence, no increment on PCO rates occurred for films thicker than 100 µm.

### 3.4. Effect of Photocatalyst Mass

**Figure 9:**
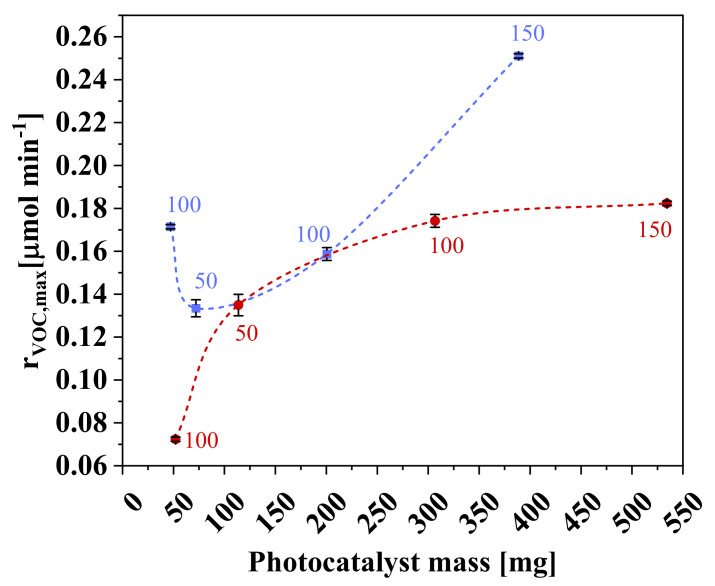
Maximum conversion rate of *n*-decane (r_VOC,max_) as a function of photocatalyst mass, with (■) and without (●) use of pore-forming agent (numbers indicate wet thickness, in microns). Conditions: irradiation intensity = 245.5 W m^−2^; *C*_VOC,feed_ = 78.8 ppm; *Q*_feed_ = 150 cm^3^ min^−1^.

Figure 9 shows the removal rate of *n*-decane as a function of photocatalyst mass. For the films without PVP, the reaction rate reached a plateau for a photocatalyst mass higher than 100 mg. This behavior is consistently observed when photocatalysts are immobilized on supports: after a certain mass amount, the particles no longer receive radiation due to agglomeration [35]. The higher mass also contributes to lesser transmissibility, which is a key point in BSI, leading to a plateau on the removal efficiency or even to a decrease [30,36].

For PVZ-4, however, even with lower mass of photocatalyst, there was a significant increase in *n*-decane removal when compared with the sample PZ-4. This result reflects two simultaneous effects: an increase in pores with PVP use and the blocking of these pores by the addition of a greater amount of photocatalyst. The conversion rate is a consequence of this balance, which had a negative outcome for the sample PVZ-3. Although the wet thickness can also affect the final morphology of the composite [37], it had a lesser effect than the use of a pore-forming agent.

In addition, a good result was obtained when lower photocatalyst concentration in the solution mixing formulation was used. As the PVZ-1 sample has a higher proportion of polymer and the light source used is completely transmissible in this material, the smaller photocatalyst amount, combined with the presence of pore-forming agent, seems to have led to better dispersion and access, promoting greater contact among irradiation, photocatalyst, and contaminant in the gas phase.

**Figure 10:**
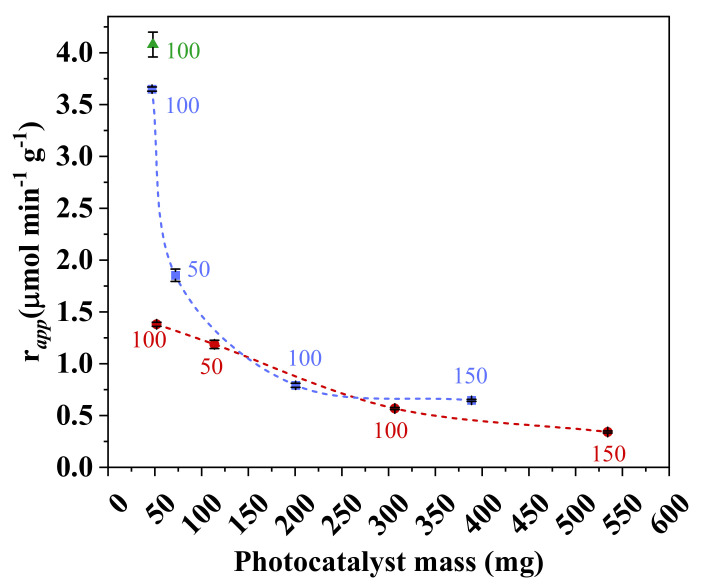
Effect of photocatalyst mass on the apparent reaction rate (*r*_app_) in ZnO composite samples with PVP (■), without PVP (●), and with APTES (▲). Conditions: irradiation intensity = 245.5 W m^−2^; *C*_VOC,feed_ = 78.8 ppm; *Q*_feed_ = 150 cm^3^ min^−1^.

Apparent reaction rate was calculated in order to compare the efficiency per gram of immobilized photocatalyst and is shown as a function of photocatalyst mass in Figure 10. In this comparison, the effects of APTES and PVP treatments were greatest for the lowest mass of photocatalyst (approximately 50 mg), where conversion efficiency was up to 2.5 times greater than that observed for the untreated sample. This confirms that the treatments provided a larger area of exposed photocatalyst.

The results of the apparent reaction rate also corroborate that the increase in *n*-decane conversion observed for sample PVZ-4 was obtained only through disproportionate photocatalyst addition. The greatest final conversion was obtained by the markedly higher photocatalyst amount in the sample of approximately 400 mg, but each particle of the photocatalyst degraded a smaller contaminant amount. This result is often seen when a large photocatalyst mass is immobilized and reflects formation of agglomerates under the preparation conditions employed.

In conclusion, the best strategy for enhancing photocatalytic efficiency is by increasing the wet thickness, which also results in an increase in the mass of photocatalyst, while retaining a low photocatalyst/polymer ratio in the formulation. In addition, the effect promoted by the use of pore-forming or compatibilizer agents is more significant in this region of low photocatalyst amount, which can also serve as a strategy for further increasing photocatalytic efficiency.

## 4. Conclusions

In this work, the preparation strategy of ZnO-based polymeric composite films was evaluated for a VOC removal in continuous flow, simulated in extreme conditions of VOC concentration and irradiation. At these circumstances, the ZnO photocatalyst in polymeric composites deactivated after a few hours of use. After characterization by FTIR-ATR, XRD, and SEM, it was not possible to state that this effect was related to photocorrosion or to the degradation of the polymeric matrix. The most likely mechanism of deactivation is related to the adsorption of CO, CO_2_, and H_2_O and formation of zinc carbonate, which was intensified by the polymer presence.

As for the effect of preparation conditions, wet thickness and photocatalyst mass of the composite films significantly influenced the process efficiency towards the removal of organics. From this analysis, it was observed that the best strategy of film preparation consisted of preparing composite films with low ZnO:PVDF ratio in formulation and high wet thickness, as well as using both pore-agent and compatibilizer additives. The reasoning of higher efficiency among these factors’ evaluation was attributed to the increase in porosity, especially by enhancing the contact with the contaminant and by the dispersion of the irradiation flow.

Composite films, such as those prepared in this study, have a relatively low manufacturing cost, easy replacement in case of deactivation, and increased catalyst stability against displacements. Despite the lower photocatalytic activity attained with the produced films when compared with the spray deposition strategy, it is important to highlight that these composite films promoted a sufficient VOC removal rate within the concentration tested. This preliminary analysis indicates that, following the strategies herein mentioned, greater conversions could be obtained for polymeric composites. Further research should focus on new materials and other operation conditions, such as front side irradiation (FSI) and turbulent regime.

## Figures and Tables

**Table 1:** Catalyst films preparation compositions and photocatalyst mass of cut samples.

Sample	Preparation Conditions					Cut Samples
	Photocatalyst		Pore Agent	Polymer	Wet Thickness	Photocatalyst Mass
	Type	Mass (g)	%wt/wt_PVDF_	Mass (g)	µm	mg
Z-1 ^a^	ZnO	0.05	-	-	-	50
P-1	-	-	6	2.25	100	-
PZ-1	ZnO	0.50	-	2.25	100	52
PZ-2	ZnO	3.00	-	2.25	50	114
PZ-3	ZnO	3.00	-	2.25	100	307
PZ-4	ZnO	3.00	-	2.25	150	534
PVZ-1	ZnO	0.50	8	2.25	100	47
PVZ-2	ZnO	2.25	8	2.25	50	72
PVZ-3	ZnO	2.25	8	2.25	100	201
PVZ-4	ZnO	2.25	8	2.25	150	389
PAZ-1	A-ZnO	0.50	8	2.25	100	48

P—polymer, V—PVP, A—APTES/CA, Z—ZnO. Samples with number 1 are samples with the same amount of photocatalyst in formulation (0.5 g) on different treatments. Samples with numbers 2, 3, and 4 are optimized formulation samples prepared with wet thickness of 50, 100, and 150 µm, respectively. ^a^ ZnO suspension sprayed directly over one side of the borosilicate slab.

**Table 2:** Beta phase fraction (F[β]) and peak intensity 101 (IXRD) for samples before and after (AT) photocatalytic oxidation tests.

Sample	F[β] (%)	F[β]-AT (%)	I_XRD_ (a.u)	I_XRD_-AT (a.u)
P-1	75.0	64.6	-	-
PZ-1	68.6	67.8	56	60
PVZ-1	71.0	72.5	85	61
PAZ-1	70.8	80.2	53	64
PVZ-4	73.1	75.4	188	146

**Table 3:** Relationship between intensities of wavelengths associated with PVDF degradation products before and after (AT) photocatalytic oxidation tests (IAT/I).

Sample	(I^AT^/I)^1752^ (a.u)	(I^AT^/I)^1711^ (a.u)	(I^AT^/I)^1613^ (a.u)	(I^AT^/I)^678^ (a.u)
P-1	0.99	0.98	0.96	0.93
PZ-1	1.07	1.00	1.16	1.22
PVZ-1	1.06	1.13	1.35	1.02
PAZ-1	1.07	1.16	1.31	1.27
PVZ-4	1.06	1.15	1.32	1.24

**Table 4:** *n*-Decane maximum conversion rate (*r_VOC,__max_*), conversion, mineralization, and apparent reaction rate (*r_app_*) using the NETmix photoreactor as well as transmittance of the catalyst films.

Sample	*r_VOC,max_* (µmol min^−1^)	Conversion (%)	Mineralization (%)	*r_app_* (µmol min^−1^ g^−1^)	Transmittance (%)
P-1	*	*	*	*	94.0
PAZ-1	0.196 ± 0.006	44 ± 3	14.3	4.1 ± 0.1	0.4
PZ-1	0.072 ± 0.001	14.9 ± 0.4	0.9	1.38 ± 0.02	1.2
PZ-2	0.135 ± 0.005	27 ± 1	11.0	1.19 ± 0.04	1.2
PZ-3	0.174 ± 0.003	37.3 ± 0.6	10.9	0.57 ± 0.01	0.0
PZ-4	0.182 ± 0.001	36.9 ± 0.6	9.0	0.34 ± 0.01	0.0
PVZ-1	0.172 ± 0.001	37 ± 1	12.5	3.65 ± 0.02	0.4
PVZ-2	0.134 ± 0.004	27 ± 1	6.4	1.85 ± 0.06	4.5
PVZ-3	0.159 ± 0.003	34.2 ± 0.5	10.1	0.79 ± 0.02	0.1
PVZ-4	0.251 ± 0.001	51 ± 2	16.9	0.65 ± 0.01	0.0
Z-1	0.450 ± 0.001	97 ± 6	84.3	8.99 ± 0.02	-

* P-1 film showed negligible removal of the *n*-decane.
